# The Application of a Non-Newtonian Fluid as a Protective Layer for a CFRP Material Subjected to Low-Energy Impact Loads

**DOI:** 10.3390/ma19030606

**Published:** 2026-02-04

**Authors:** Piotr Arkuszyński, Marek Rośkowicz, Angelika Arkuszyńska

**Affiliations:** Faculty of Mechatronics, Armament and Aerospace, Military University of Technology, 00-908 Warsaw, Poland; marek.roskowicz@wat.edu.pl (M.R.); angelika.arkuszynska@wat.edu.pl (A.A.)

**Keywords:** composite material, CFRP, non-Newtonian fluid, impact loads, impact

## Abstract

One of the key challenges in using CFRP (Carbon Fiber Reinforced Polymer) structures is their susceptibility to low-energy impact damage, often indicated as barely visible impact damage (BVID). Such defects are difficult to detect and can compromise structural integrity. This study investigates the use of immobilized non-Newtonian fluids (NNF) as protective layers for CFRP composites subjected to low-energy impacts. Experimental tests were carried out with an Instron 9440 drop-weight impact tower (impact energy range 5–40 J) and high-speed imaging, comparing NNF coatings with rubber-based, caoutchouc-based, and spray-based protective layers. Non-destructive evaluation using computed tomography confirmed that NNF coatings dissipate impact energy through shear-thickening behavior, reducing delamination while preserving clear visual indicators of the impact site. Furthermore, the study assessed post-impact fatigue bending performance, revealing that the inclusion of NNF—either as an outer layer or as part of a sandwich structure—significantly enhanced the residual fatigue strength of the composites. Moreover, NNFs inherently preserve visible traces of penetration, thereby improving the detectability of impact locations through both unaided visual inspection and advanced imaging modalities such as computed tomography. In addition to external coatings, NNF was applied as a core in sandwich structures, demonstrating improved impact resistance compared to monolithic CFRP laminates and conventional CFRP–foam sandwiches. The protective performance was found to depend on fluid thickness and threshold shear rates required for viscosity transition, indicating that thicker layers do not always provide superior protection.

## 1. Introduction

Carbon-fiber-reinforced polymer (CFRP) composites have become increasingly prevalent across a broad spectrum of engineering applications, ranging from consumer goods such as sporting equipment to advanced automotive and aerospace structures. Their widespread utilization is driven by a combination of advantageous properties, including a high stiffness-to-weight ratio, excellent fatigue resistance, and inherent immunity to electrochemical corrosion. These characteristics contribute to significant weight reduction and enhanced durability, which are critical for performance and efficiency in demanding sectors [1,2,3].

Despite these benefits, CFRP composites exhibit a notable vulnerability to low-energy impact damage, which poses a serious challenge to their structural integrity and long-term performance [4]. Such impacts, often resulting from tool drops, bird strikes, debris impact during service, may induce barely visible impact damage (BVID) [5,6]. This damage is particularly insidious as it is difficult to detect using conventional visual inspection, necessitating advanced non-destructive testing (NDT) techniques for identification. Importantly, BVID can significantly compromise the residual fatigue strength of CFRP components, increasing the risk of premature failure under cyclic loading conditions [7,8].

To mitigate these risks, protective surface layers have traditionally been employed to absorb or dissipate impact energy and to enhance the detectability of damage. Common protective solutions include elastomeric coatings and paint-based systems that primarily rely on elastic deformation and localized energy absorption mechanisms. However, these conventional approaches often present limitations, such as insufficient energy dissipation efficiency and limited enhancement of damage visibility, particularly for subtle internal defects [9,10,11]. As an alternative and more advanced approach, recent studies have proposed the use of ultra-thin stainless-steel foils integrated directly with CFRP as protective surface layers, enabling impact energy redistribution and damage suppression without the need for an additional adhesive film [12].

Recent research efforts have investigated foam-based sandwich cores as an alternative means of impact mitigation. While these cores improve energy absorption capacity, they still fall short in facilitating damage detectability and may add undesirable weight or complexity to structural components [13,14]. In response to these limitations, attempts have been made to develop sandwich composite structures incorporating non-Newtonian fluids as core materials. Such solutions aim to combine lightweight structural design with adaptive, strain-rate-dependent impact resistance. Experimental investigations have demonstrated that the introduction of a shear-thickening core can significantly enhance impact performance. In particular, structures with a thin non-Newtonian fluid core exhibited a substantial increase in absorbed impact energy, whereas configurations with a thicker fluid core effectively reduced the extent of damage propagation, especially on the rear surface of the composite panels. These findings indicate that non-Newtonian fluid cores may offer a promising alternative to conventional foam cores, improving both impact energy dissipation and damage suppression mechanisms without excessive structural mass increase [15].

In this context, non-Newtonian fluids (NNFs) have emerged as a class of materials with significant potential as adaptive protective materials due to their unique rheological behavior. Characterized by a shear-thickening response, NNFs exhibit a marked increase in viscosity when subjected to high strain rates, effectively transitioning from a fluid-like to a solid-like state under impact loading [16]. This dynamic change allows NNFs to dissipate impact energy more efficiently than conventional materials while maintaining flexibility under normal conditions [17].

Beyond CFRP materials, the broader literature on NNF–based protective systems, for example, in fabric composites and personal protective equipment, shows that the involvement of non-Newtonian fluids can enhance inter-fiber friction, improve energy dissipation, and reduce penetration depth under impact or ballistic loading [18]. However, the observed effects are strongly dependent on fluid composition, particle concentration, and structural configuration. Furthermore, some studies indicate that fluid–structure interaction mechanisms may, in specific laminate protection circumstances, favor shear-thinning rather than shear-thickening behavior. This suggests that the optimal rheological profile for impact mitigation is application-specific and depends on loading conditions as well as geometric limitations [19].

The present study aims to systematically evaluate the efficacy of immobilized NNFs as protective elements for CFRP composites, considering two principal configurations: external coatings applied to monolithic laminates and as core materials integrated into sandwich structures. Previous studies investigating the use of non-Newtonian fluids (NNFs) as external protective layers have primarily focused on applying the material in its thin liquid form, typically using a roller, which resulted in a non-permanent coating. Nevertheless, these investigations demonstrated that even such a thin layer was capable of absorbing up to 45% of the impact energy during low-energy impact events [20]. In contrast, non-Newtonian fluids have predominantly been studied as fiber-impregnating agents rather than as discrete protective layers [21,22], highlighting the novelty of the approach adopted in this work. Here, a comprehensive experimental methodology is employed, combining instrumented low-velocity impact testing, high-speed imaging to capture the dynamic response, and computed tomography for detailed damage morphology characterization. Special emphasis is placed on understanding the influence of fluid layer thickness and the associated viscosity transition thresholds, which are critical parameters governing the protective performance of NNF layers.

This investigation employs a comprehensive experimental approach combining instrumented low-velocity impact testing, high-speed imaging for capturing dynamic response, and computed tomography for detailed damage morphology characterization. Emphasis is placed on understanding the influence of fluid layer thickness and the associated viscosity transition thresholds, which are critical parameters governing the protective performance of NNF layers.

In contrast to previous studies where non-Newtonian fluids were mainly used as additives (e.g., fiber sizing agents) or as temporary surface coatings, the novelty of the present work lies in the application of an immobilized non-Newtonian fluid as an independent and permanent structural element. The NNF is implemented in two configurations: as an external protective layer and as a core material within a sandwich structure. Importantly, the fluid is mechanically confined without the use of additional adhesive modifiers or rheological stabilizers, which distinguishes this concept from earlier approaches relying on chemical integration with the composite matrix. This design moves beyond laboratory-scale demonstrators toward a structurally integrated solution that, following further development, has the potential for application in aerospace structures rather than remaining limited to experimental proof-of-concept systems.

Additionally, the study includes an evaluation of residual mechanical properties following impact, with particular emphasis on fatigue strength degradation. Such evaluation is essential to determine the long-term viability of NNF-based protection systems in demanding engineering applications where both impact resistance and durability under cyclic loading are paramount. To date, however, most research on non-Newtonian fluid applications in composite protection has focused predominantly on single-impact response or quasi-static behavior, while the post-impact fatigue performance of structures incorporating immobilized NNF layers has remained largely unexplored. In particular, the influence of a permanently confined NNF layer on damage propagation mechanisms, stiffness degradation, and fatigue life under cyclic loading conditions has not been systematically investigated.

Findings from this research are intended to support the development of advanced lightweight composite structures featuring improved damage tolerance and greater service reliability.

## 2. Materials and Methods

**Figure 1:**
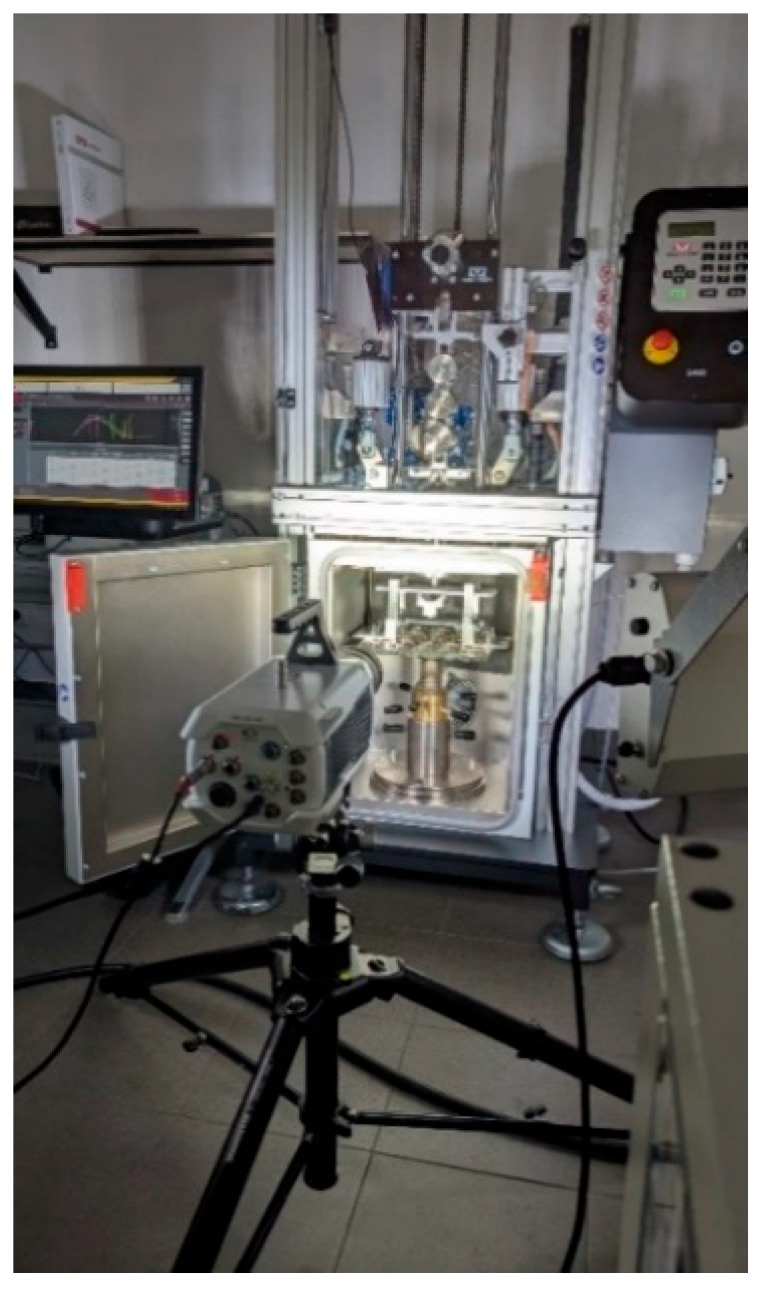
Impact test bench (including high-speed camera) [23].

The tested material was an orthotropic composite, 3.8 mm thick, composed of 26 unidirectional layers arranged in a [0°]_26_ configuration. It was manufactured using a hydraulic press process, based on CC 160 g/m^2^ carbon fabric (Kordacarbon, Strážnice, Czech Republic) and impregnated with LR 285 epoxy resin cured with LH 286 hardener (MGS, Erkelenz, Germany). In order to reduce the risk of residual stress accumulation, the specimens were precisely cut to dimensions of 250 mm × 60 mm using a WaterJet cutting technique. Low-velocity impact tests were carried out using an Instron 9440 drop-weight impact tower (Instron, Norwood, MA, USA), which ensures high accuracy and repeatability of the impact loading process. Each impact was recorded using a Phantom T3610 high-speed camera (Vision Research, Wayne, NJ, USA), operating at frame rates of up to 772,000 frames per second, enabling detailed analysis of the material’s dynamic response during impact and during the early stage of the post-impact response. The experimental setup used for impact tests is presented in Figure 1.

**Figure 2:**
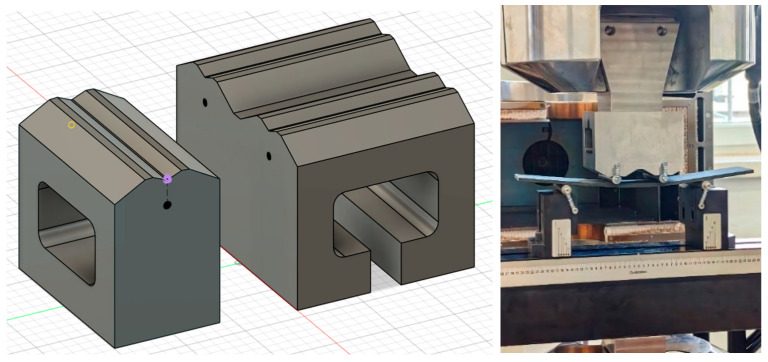
Design and implementation of a setup for measuring fatigue bending strength following impact loading.

To evaluate the fatigue performance after impact, a dedicated experimental setup was designed (using Fusion 360 software v.2.0.21550) and constructed (Figure 2), enabling direct assessment of post-impact fatigue behavior under controlled loading conditions.

Fatigue tests were conducted using an MTS servo-hydraulic testing machine (MTS Systems Corporation, Eden Prairie, MN, USA), which provided precise control of loading parameters and accurate determination of the number of cycles to failure, serving as a reliable indicator of the residual fatigue performance of the CFRP specimens.

The ultimate destructive force of an undamaged reference specimen was determined from static tests of three samples, yielding an average value of 1.942 kN. Applying a safety factor of 1.5, the fatigue load level was set to 1.295 kN. The undamaged reference specimen has currently completed 900,000 load cycles, and the test remains ongoing at the time of writing.

Fatigue loading was applied at a frequency of 3 Hz, ensuring stable testing conditions while preventing excessive specimen heating that could otherwise affect fatigue strength.

**Figure 3:**
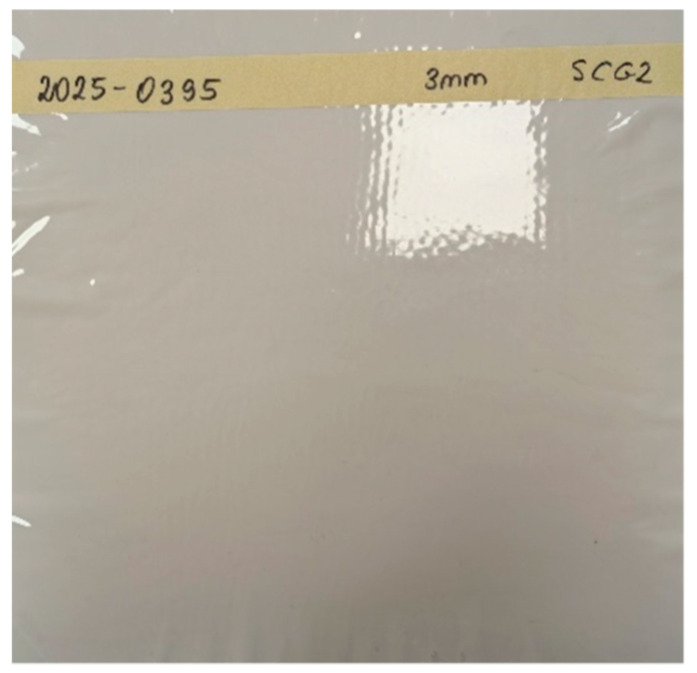
Sheet of immobilized non-Newtonian fluid.

The immobilized non-Newtonian fluid, provided by SmartFluid (Smart Fluid S.A., Warsaw, Poland), was applied directly onto the specimens without the use of any additional adhesive layer. The material was supplied in the form of ready-to-use sheets positioned between two protective polymer films. In this form, the fluid exhibits a putty-like consistency, allowing it to adhere readily to flat surfaces. A representative ready-to-use fluid sheet is shown in Figure 3. The commercial name of the applied material is Smart Cerami Gel 2 (SCG2). According to the manufacturer’s specifications, the material has a density of 1.55 g/cm^3^ and a dynamic viscosity in the range of 500–700 Pa·s. SCG2 (Smart Fluid S.A., Warsaw, Poland) is a patented material developed by SmartFluid, a company specializing in advanced material technologies and functional protective solutions.

**Figure 4:**
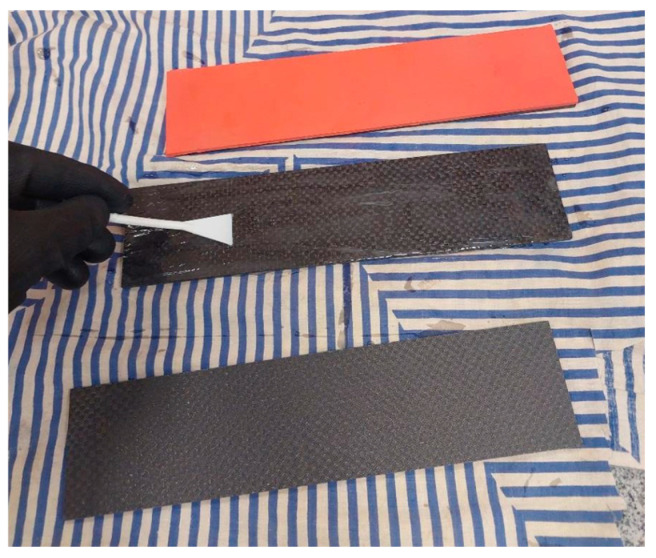
Specimens during the application of external rubber and caoutchouc layers.

For comparative purposes and to better understand the energy dissipation mechanisms of the non-Newtonian fluid (NNF), reference specimens including coatings of rubber, caoutchouc (bonded using a two-component epoxy adhesive, Technical), and Raptor protective paint were also investigated. The process of applying external protective layers in the form of rubber and caoutchouc is presented in Figure 4.

The protective paint coating was directly applied to the specimen surface using an atomizer supplied by Raptor (U-Pol Ltd., Wellingborough, UK).

It is worth noting that the surfaces of all specimens were previously degreased with isopropyl alcohol (Merck KGaA, Darmstadt, Germany) prior to the application of the external protective layers.

**Figure 5:**
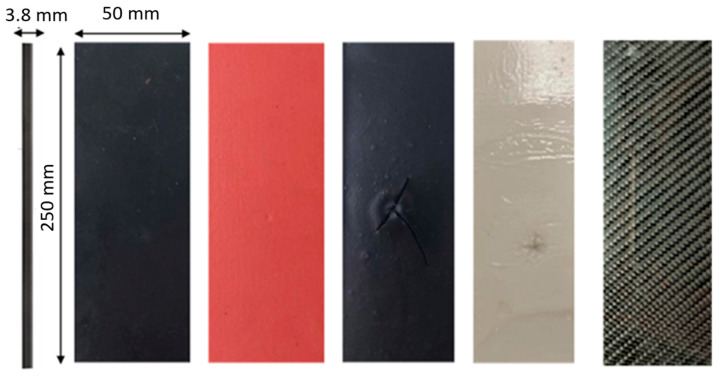
Specimens used in the research.

A CFRP composite specimen without a protective layer, as well as specimens with all protective layers applied, are presented in Figure 5. From left to right (flatwise orientation), the specimens are coated with rubber, caoutchouc, protective paint, a non-Newtonian fluid layer, and finally an uncoated reference specimen.

**Figure 6:**
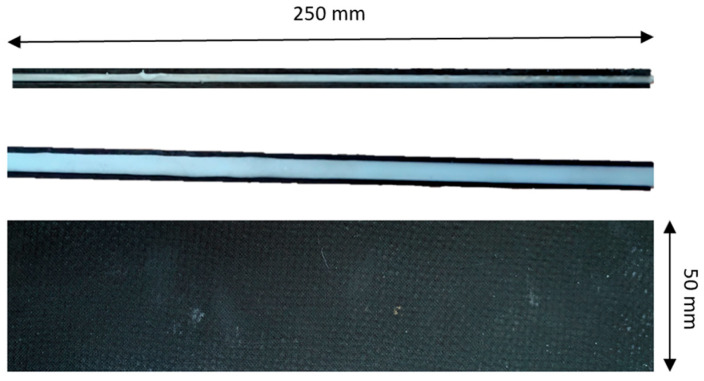
Representative sandwich-type specimens.

In addition to the investigation of external protective layers, the experimental campaign was extended to include sandwich-type composite configurations incorporating an immobilized non-Newtonian fluid as the core material. For these specimens, the non-Newtonian fluid was placed between two CFRP face sheets, forming a sandwich structure designed to evaluate the impact mitigation capability of the fluid when employed as an internal energy-absorbing medium. Sandwich-type specimens with a non-Newtonian fluid core of 2 mm and 5 mm thickness are presented in Figure 6.

As a reference configuration, sandwich composites with an aerospace-grade foam core of identical thickness were manufactured and tested under the same impact conditions. This comparative approach enabled a direct assessment of the effectiveness of the non-Newtonian fluid core relative to conventional lightweight core materials commonly used in aerospace sandwich structures.

A GE Phoenix v/tome/x m 300 computed tomography (CT) system (Waygate Technologies, Wunstorf, Germany), equipped with an X-ray source capable of operating at up to 300 kV/500 W, was employed to assess the post-impact condition of the composite specimens. The CT scans were conducted using the following parameters: X-ray tube voltage of 120 kV, current of 120 μA, projection counts ranging from 360 to 1440, and beam filtration utilizing a 0.5 mm Cu + 0.5 mm filter combination to assess internal damage morphology following low-velocity impacts.

## 3. Results and Discussion

### 3.1. NNF as an External Protection Layer

**Figure 7:**
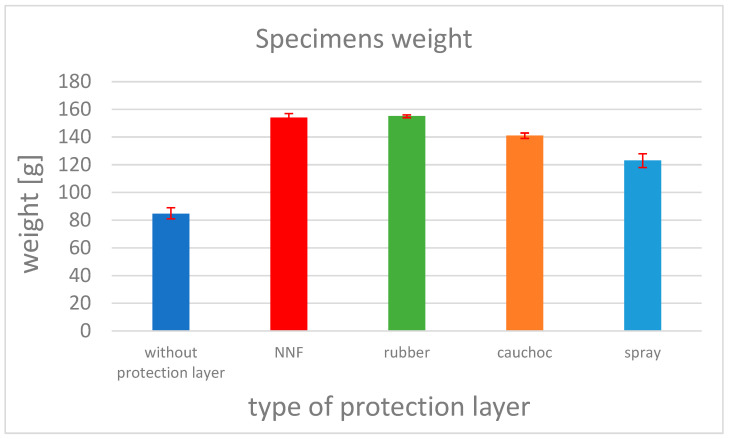
The influence of the applied outer layer on mass increase.

All specimens were weighed to evaluate the influence of the applied outer layer on mass increase. The differences between the specimens were negligible, except for the spray layer, where the weight change was significantly lower than for the other layers. The results are presented in Figure 7.

**Figure 8:**
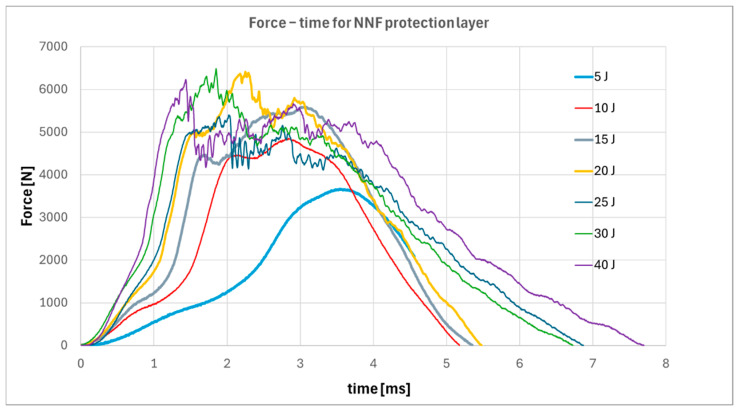
Force–time responses for specimens with a protective layer based on a non-Newtonian fluid.

Thanks to the use of an instrumented impactor with a spherical tip geometry compliant with ISO 8256, it was possible to register force-time responses during low-velocity impact tests [24]. All specimens were tested within an impact energy range of 5 to 40 J, which corresponds to typical damage scenarios encountered during aircraft service conditions. The force-time response recorded for the specimen with a non-Newtonian fluid-based protective layer is presented in Figure 8.

Based on the analysis of the force-time responses, it can be observed that at low impact energy levels (5 J), the non-Newtonian fluid provides sufficient damping to completely absorb the applied energy, as indicated by the lack of rebound in the force signal. However, once the impact energy exceeds 20 J, initial indications of damage propagation within the material begin to appear, suggesting this value as a threshold for the protective capability of the applied layer. Sudden changes in the force response prior to reaching the peak load are commonly linked to the failure of the surface ply impacted by the striker. A decline in force following the maximum value typically indicates the initiation of delamination between layers. In cases where the force signal exhibits large fluctuations and prolonged contact between the impactor and the specimen is observed, full perforation of the composite can be assumed (Figure 4) [25,26,27].

**Figure 9:**
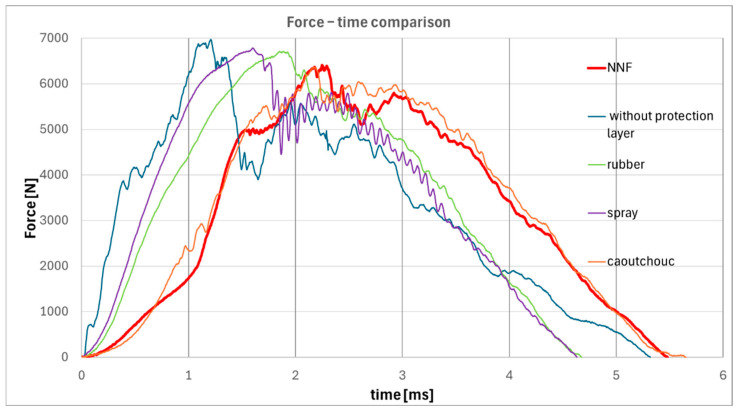
Force–time responses for specimens with all types of protective layers.

The force-time responses of specimens with all types of external protective layers were compared at an impact energy of 20 J, which corresponds to the maximum threshold value identified for non-Newtonian fluids. The results are shown in Figure 9.

Detailed analysis of the force-time responses reveals that the mechanical behavior of the specimen protected with a non-Newtonian fluid layer most closely resembles that of the specimen with a caoutchouc-based protective layer. In particular, the non-Newtonian fluid demonstrates markedly lower oscillation amplitudes throughout the loading and unloading phases compared to the other protective systems examined. Furthermore, no abrupt force drops are observed, with the exception of the initial stage of impact corresponding to the penetration of the outer layer. Such drops, commonly associated with matrix cracking or delamination events in composite structures, are absent in the subsequent stages of the loading process. This observation strongly suggests that the non-Newtonian fluid provides superior energy dissipation, effectively reducing stress wave propagation into the underlying CFRP laminate.

**Figure 10:**
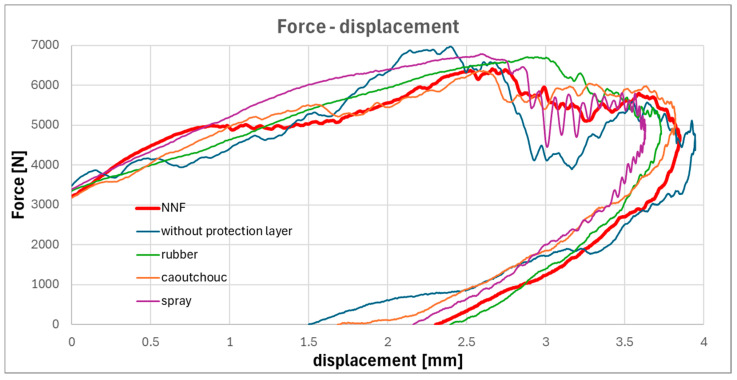
Force–displacement responses for specimens with all types of protective layers.

The mechanism of energy dissipation resulting from viscosity variations in the non-Newtonian fluid is more clearly observable in the force-displacement responses. A comparative analysis of these responses for all investigated external protective layers is presented in Figure 10.

Analysis of the force-displacement response indicates that the mechanical behavior of the non-Newtonian fluid layer most closely resembles that of rubber up to the point of maximum displacement. Beyond this point, a transition in the response is observed: initially, the behavior remains comparable to caoutchouc, but in the final stage of the impact event, it increasingly resembles that of the spray-based protective layer. This progressive transformation in response is attributed to the viscosity change of the non-Newtonian fluid under dynamic loading conditions. Notably, the increase in viscosity does not occur instantaneously but rather develops throughout the impact.

**Figure 11:**
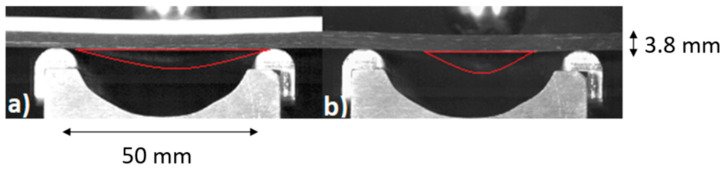
Specimens at maximum displacement during impact. (**a**) With NNF protection layer. (**b**) Without a protection layer.

These observations are confirmed by high-speed camera recordings, which validate the staged nature of the transition of the viscosity and its influence on the energy dissipation mechanism. During the impact, the internal structure of the non-Newtonian fluid undergoes a pronounced transformation resulting from shear-induced viscosity changes. In an unprotected specimen, the absence of a damping medium leads to a highly localized, point-type puncture, concentrating stress in a narrow region and promoting rapid fiber fracture. Conversely, specimens protected with the non-Newtonian fluid exhibit a transition from localized to distributed, surface-type puncture behavior. This transition allows the impact energy to be dispersed over a significantly larger area of the laminate, engaging a greater number of reinforcing fibers in the energy absorption process. As a result, the local stress peaks are substantially reduced, preventing them from exceeding the critical thresholds required to initiate catastrophic fiber breakage or matrix cracking. The differences in puncture morphology between unprotected CFRP specimens and those protected with a non-Newtonian fluid at the point of maximum displacement are presented in Figure 11.

The tomographic analyses confirm that the non-Newtonian fluid layer offers the most effective protection for CFRP specimens impacted at an energy level of 20 J. In these specimens, no evidence of delamination or fiber fracture was detected, indicating a complete suppression of critical damage mechanisms.

**Figure 12:**
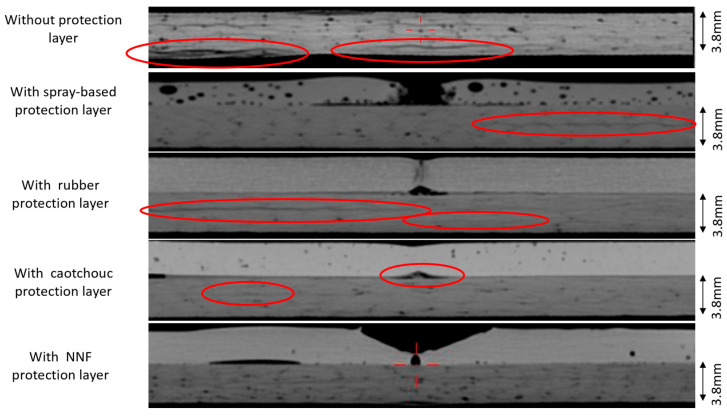
Post-impact tomograms of all specimens subjected to 20 J impact energy.

In contrast, all other protective configurations exhibited varying degrees of internal damage. The most prevalent failure modes included delamination between plies, interlaminar cracking, and localized fiber pull-out, particularly pronounced in specimens protected with the caoutchouc layer at the impact contact zone. These findings highlight the superior energy-dissipation capability of the non-Newtonian fluid, effectively mitigating stress concentrations and preventing structural degradation of the laminate. A compilation of tomographic images of all specimens, with the identified defects highlighted, is presented in Figure 12**,** where the defects are marked with red ovals.

**Figure 13:**
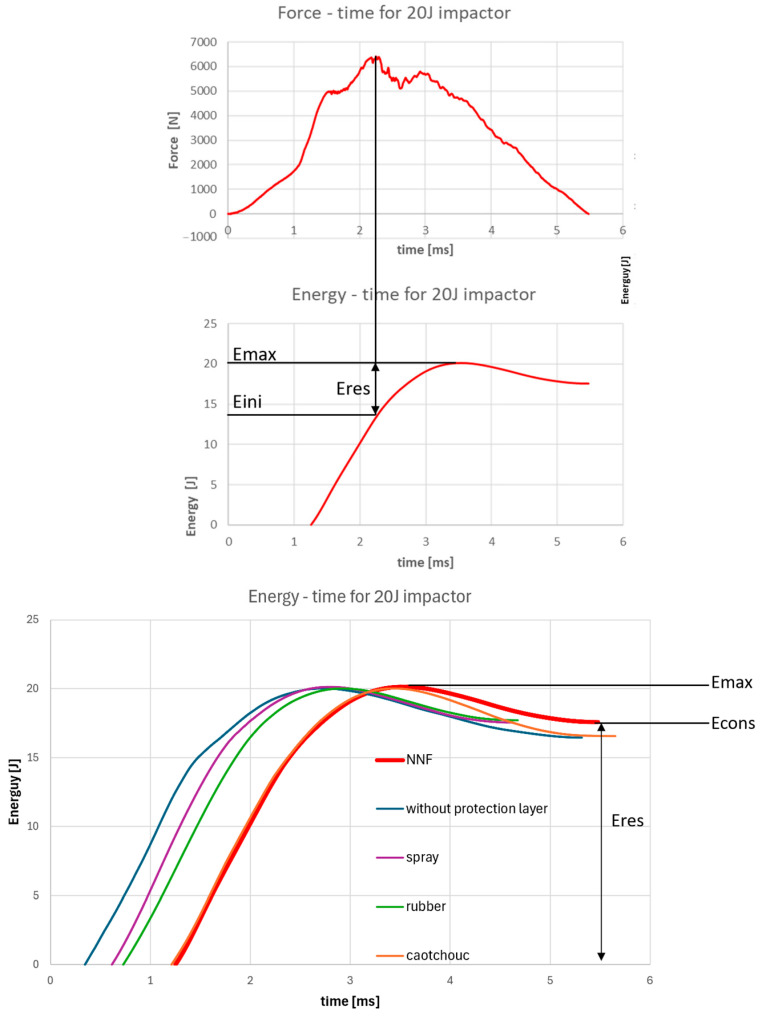
Standard graphical representations of Eres.

In the available literature, two primary approaches are reported for estimating the energy level at which damage to the material occurs (commonly referred to as Eres). The first approach defines Eres as the difference between the energy corresponding to the maximum force recorded by the impact hammer (Eini) and the total kinetic energy of the impactor. The second approach interprets Eres as the difference between the maximum energy and the constant energy after impact [28]. Both approaches are shown in Figure 13.

However, when applied to non-Newtonian protective layers, both methodologies become inadequate. Neither approach accounts for the energy consumed in damaging the protective layer itself or for the energy dissipation associated with the increase in viscosity of non-Newtonian fluids under dynamic loading. As a consequence, conventional indicators may misleadingly suggest poor protective performance of the non-Newtonian layer, which contradicts the experimental findings presented in the previous section, where this material demonstrated superior impact protection performance. Conventional Eres indicators are shown in Table 1.

Estimating the extent of impact-induced damage alone does not enable an unambiguous assessment of the corresponding degradation in the material’s fatigue strength. Therefore, post-impact fatigue testing is essential to accurately quantify the residual load-carrying capability, and in the present study, such tests were conducted in accordance with the methodology described in Section 2.

**Figure 14:**
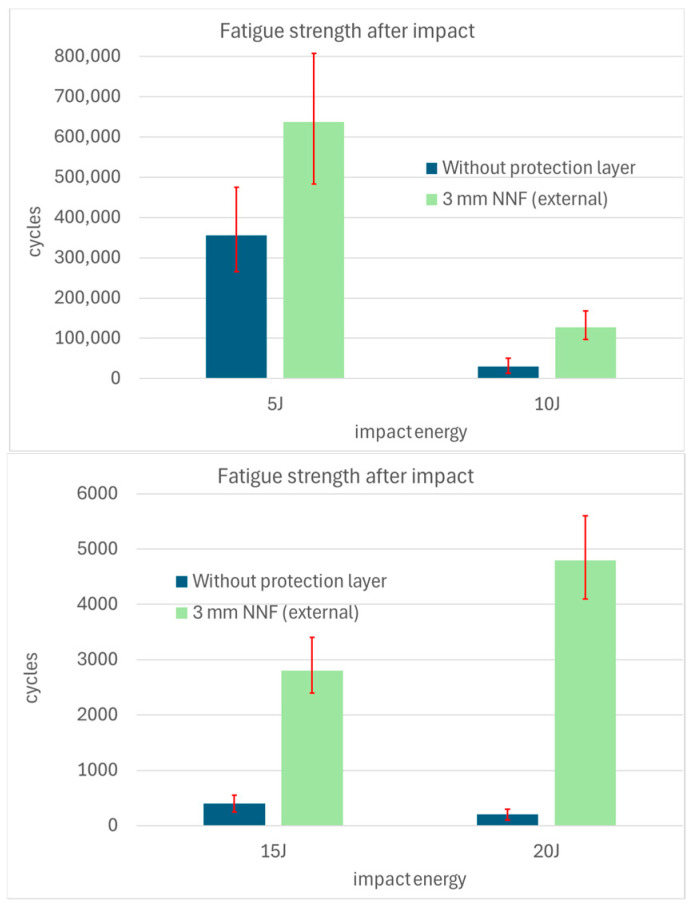
Fatigue test results (after impacts).

The average fatigue test results, obtained from three specimens per impact energy level and comparing CFRP material without a protective layer and specimens coated with a non-Newtonian fluid outer layer, are presented in Figure 14.

At the lowest impact energy of 5 J, the fatigue life of the specimen with the protective layer nearly doubled, increasing from 355,000 to 638,000 cycles. At 10 J, this improvement was approximately fourfold, with cycle counts rising from 29,000 to 127,000. For 15 J, the fatigue life increased sevenfold, from 400 to 2800 cycles. At the highest tested energy of 20 J, specimens without a protective layer failed almost immediately, whereas those with the non-Newtonian fluid layer endured an average of 4800 cycles before failure.

**Figure 15:**
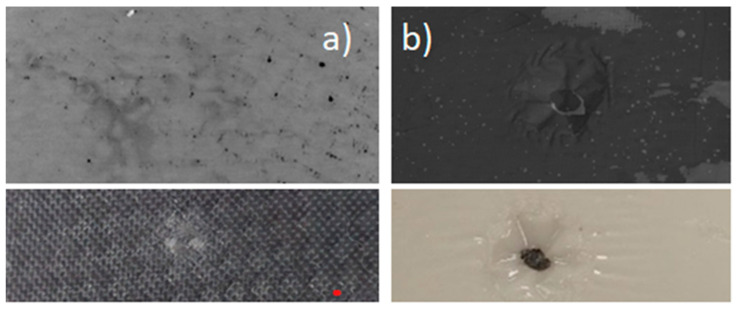
Impact location tomogram and unaided eye view. (**a**) Without protection. (**b**) With an NNF protection layer.

An additional advantage of applying a non-Newtonian fluid as an external protective layer for CFRP composite materials is its ability to facilitate the identification of the impact location. Low-energy impacts can induce barely visible impact damage (BVID), which poses a significant hazard in aerospace applications. In many cases, the time required to locate the damaged area exceeds the duration of the subsequent non-destructive inspection itself. In specimens with the non-Newtonian protective layer, the impact site is more easily identifiable due to localized traces of penetration formed within the layer. This effect results from the high viscosity of the fluid during impact, which preserves a distinct imprint of the contact area. The differences in the detectability of the impact sites for CFRP specimens with and without the non-Newtonian protective layer are presented in Figure 15, both in visual inspection with the unaided eye and in tomographic imaging, confirming the enhanced visibility of damage locations when the protective layer is applied.

### 3.2. NNF as a Core in Sandwiches

The second configuration, implementing a non-Newtonian fluid as a protective medium, involved its placement within the core of a sandwich composite structure. In this design, the outer CFRP skins were manufactured with half the thickness of those used in the previous test series, consisting of 13 plies. Two variants of the non-Newtonian fluid layer were examined, with thicknesses of 2 mm and 5 mm, respectively. For comparative purposes, reference specimens included sandwich panels with a low-density foam core, with the same thicknesses as NNF, and a standard monolithic specimen from the previous test series.

**Figure 16:**
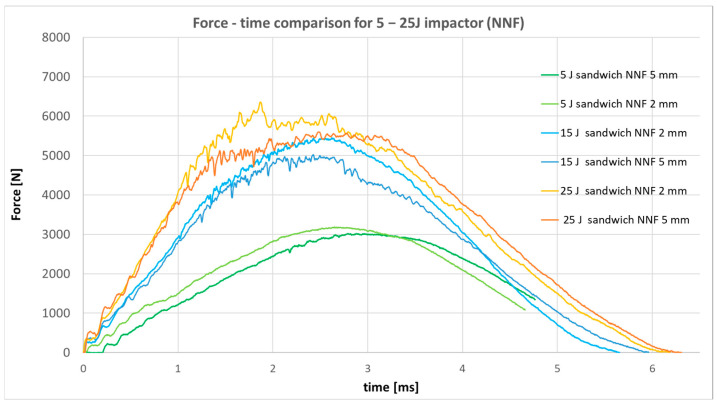
Force–time responses for specimens with a non-Newtonian fluid core.

The Instron 9440 drop-weight impact tower was subsequently used to record force-time responses during low-velocity impact tests. All specimens were examined within an impact energy range of 5–25 J. The force-time response recorded for the specimen with a non-Newtonian fluid-based core is presented in Figure 16.

**Figure 17:**
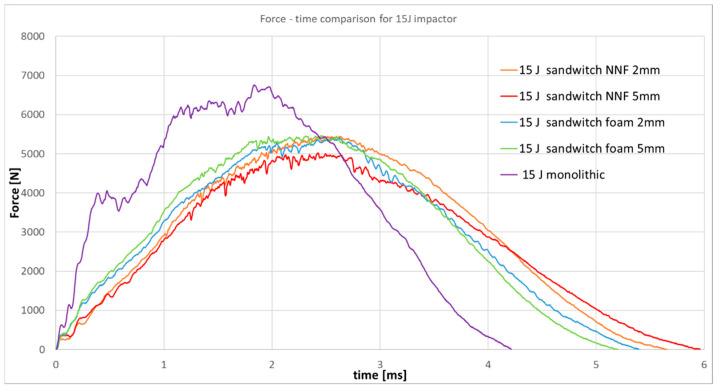
Force–time responses for 15 J impact energy.

**Figure 18:**
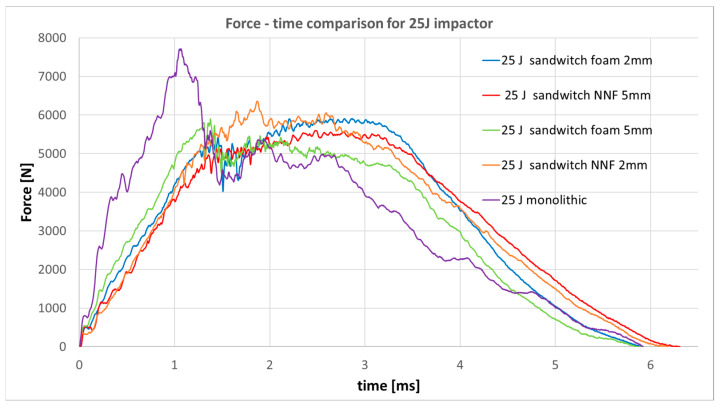
Force–time responses for 25 J impact.

Analysis of the recorded force-time curves indicates that, for the lowest impact energy of 5 J, the non-Newtonian fluid layer fully dissipates the impact energy, preventing the onset of structural damage. At higher energy levels, namely 15 J and 25 J, minor oscillations are observed in the response curves, suggesting the initiation of defects within both the CFRP skins and the non-Newtonian fluid core. An interesting correlation is observed: specimens incorporating the thinner fluid layer exhibit reduced oscillation amplitudes compared to those with the thicker layer, implying superior protective efficiency despite the lower fluid volume. Accordingly, a comparative analysis with specimens included sandwich panels with a low-density foam core, with the same thickness as the NNF core, and a standard monolithic specimen was carried out for 15 J impact (Figure 17) and 25 J impact (Figure 18).

In the force-time graph corresponding to 15 J and 25 J impact energy, it is evident that the non-Newtonian fluid layer provides superior protection compared to conventional aerospace-grade foam. The recorded response exhibits reduced oscillation amplitudes, while the peak force (maximum recorded value) is lower than that observed for the standard CFRP specimen. However, the force decreases more rapidly, reaching levels below those of the sandwich configuration. This behavior can be attributed to the fact that, in the sandwich, the initial response is governed exclusively by the outer layer, which is only half as thick as a monolithic specimen, and the principal energy dissipation occurs later in the event, within the core material.

**Figure 19:**
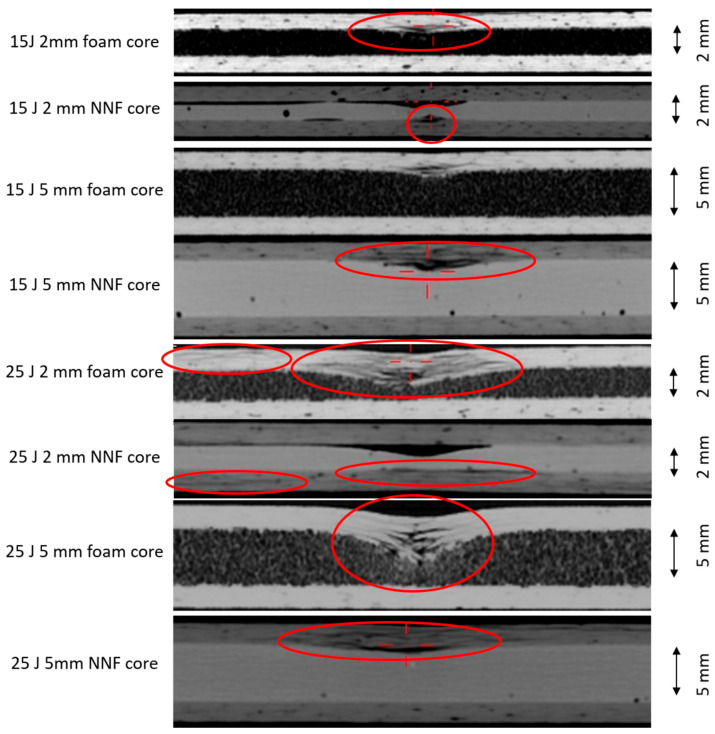
Post-impact tomograms of sandwich specimens subjected to 15 J and 25 J impact energy.

A series of tomographic examinations confirmed that smaller internal defects occur in the sandwich specimens incorporating a thinner non-Newtonian fluid core. Moreover, a distinct variation in damage morphology is observed. In specimens incorporating the thinner fluid layer, both laminate layers exhibit damage, but to a reduced degree compared to the single outer laminate layer in the sandwich specimens containing a 5 mm fluid layer. A compilation of tomographic images of all specimens, with the identified defects highlighted, is presented in Figure 19.

**Figure 20:**
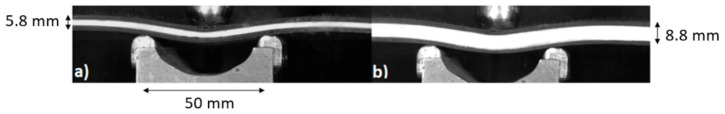
Sandwich specimens at maximum displacement during 25 J energy impact. (**a**) With a 2-mm NNF core. (**b**) With a 5-mm NNF core.

Based on the high-speed camera recordings, it can be concluded that a thinner fluid layer leads to lower stiffening of the outer CFRP laminate layer in contact with the striker, thereby facilitating more efficient energy dissipation during impact [29,30]. The differences in impact energy dissipation between specimens characterized by different non-Newtonian fluid core thicknesses are illustrated in Figure 20.

The second factor is related to the behavior of non-Newtonian fluids, which exhibit both minimum and maximum threshold values for viscosity change. In this case, the minimum force (shear rate) threshold necessary to trigger the viscosity change was not achieved in the specimens with the thicker fluid layer, which explains their reduced protective performance [31,32,33,34,35,36].

## 4. Conclusions

The implementation of non-Newtonian fluids (NNF) as a protective layer in CFRP composite structures significantly enhances their resistance to low-energy impacts. The protective mechanism is attributed to the viscosity change of the fluid under dynamic loading, which facilitates partial dissipation of impact energy and reduces the severity of structural degradation.

These findings are consistent with the theoretical framework of shear-thickening fluids, in which viscosity increases under high strain rates, leading to a temporary solid-like behavior that enhances protective performance. This mechanism, combined with the observed stress redistribution, underscores the potential of non-Newtonian fluid layers as effective protective systems for CFRP structures subjected to low-velocity impacts.

When applied as an external protective layer, NNF layers reduce the extent of delamination in CFRP laminates despite contributing the smallest stiffness increase compared to conventional protective materials such as paint, rubber, or caoutchouc. Furthermore, such coatings improve the detectability of impact locations, as the fluid preserves visible traces of penetration, aiding both visual and NDT inspection.

Finally, the protective properties of NNF result in a substantial improvement in post-impact fatigue bending strength, indicating its potential for use in aerospace and automotive structures where low-energy impact resistance and damage tolerance are critical.

The use of NNF within sandwich structures as a core material also improves impact resistance compared to monolithic CFRP laminates and conventional CFRP-foam sandwiches. However, this protective effect is strongly influenced by the fluid layer thickness and the threshold shear rate required for viscosity transition; consequently, thicker fluid layers do not necessarily provide superior protection.

Additionally, both external and core applications of NNF extend the contact time between the impactor and the specimen, enabling less destructive stress distribution throughout the composite.

## Figures and Tables

**Table 1:** Conventional Eres indicators of all specimens.

20 J Impact				
specimen type	F_max_ [N]	E_ini_ [J]	E_max_ [J]	E_res_ [J]
without a protection layer	6974.52	11.93	20.02	8.09
rubber	6712.23	14.97	20.01	5.04
spray	6787.70	14.19	20.13	5.98
caoutchouc	6369.51	12.80	20.01	7.21
NNF	6413.26	13.47	20.13	6.67

## Data Availability

The original contributions presented in this study are included in the article. Further inquiries can be directed to the corresponding author.
